# Supplementary material for: Quantifying 3D Strain in Scaffold Implants for Regenerative Medicine
Source: Materials (Basel). 2020 Sep 3;13(17):3890. doi: 10.3390/ma13173890 (PMC7504351; doi:10.3390/ma13173890)
Supplement: Supplementary file 1 [file materials-13-03890-s001.pdf]

materials

Supplementary Materials

Quantifying 3D Strain in Scaffold Implants for Regenerative Medicine

Jeffrey N. Clark [1,2], Saman Tavana [1], Agathe Heyraud [2], Francesca Tallia [2], Julian R. Jones [2], Ulrich Hansen [1] and Jonathan R.T. Jeffers [1,*]

1 Department of Mechanical Engineering, Imperial College London, South Kensington Campus, London SW7 2AZ, UK; j.clark14@imperial.ac.uk (J.N.C.); s.tavana17@imperial.ac.uk (S.T.); u.hansen@imperial.ac.uk (U.H.)
2 Department of Materials, Imperial College London, South Kensington Campus, London SW7 2AZ, UK; agathe.heyraud13@imperial.ac.uk (A.H.); f.tallia@imperial.ac.uk (F.T.); julian.r.jones@imperial.ac.uk (J.R.J.)
* Correspondence: j.jeffers@imperial.ac.uk; Tel.: +44-(0)20-7594-5471

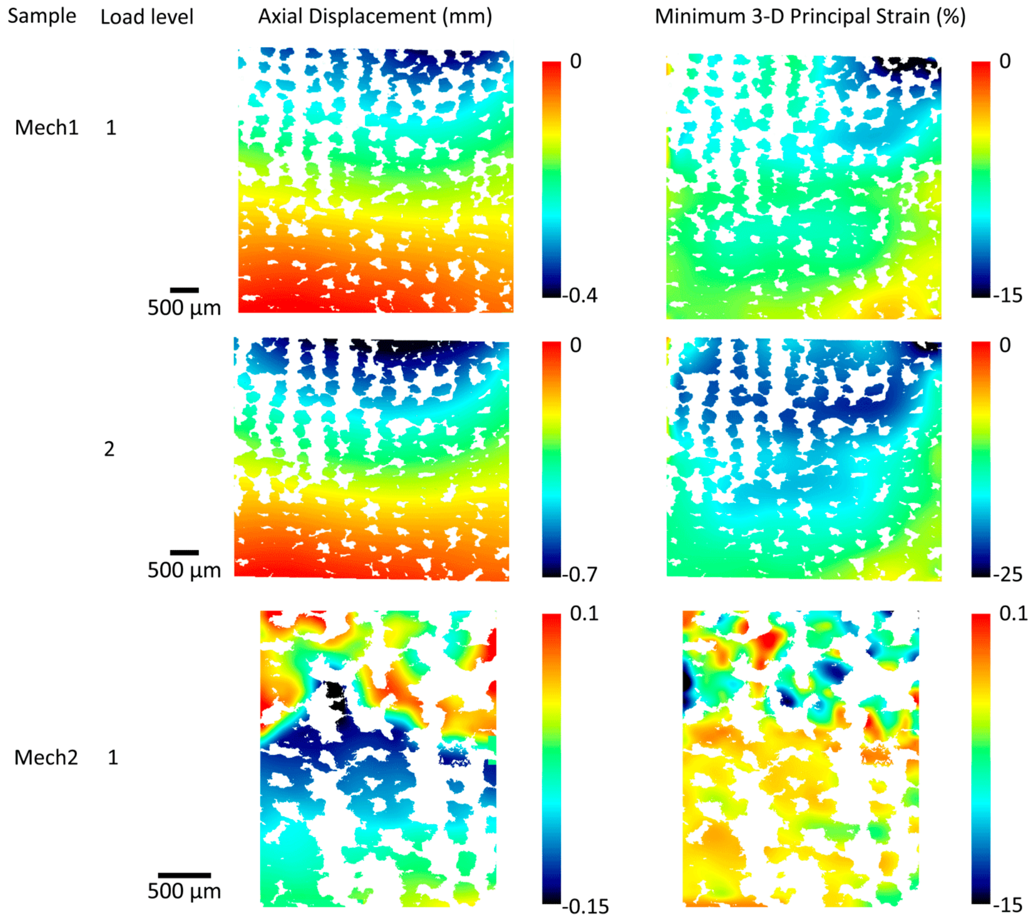

Figure S1. Displacement and strain calculated for cubic hybrid implants intended for mechanical testing and device development, calculated using DVC. Shown here is one vertical slice through the centre of each sample. Note that the scales for the Mech2 sample include positive values for both axial displacement and minimum 3D principal strain.

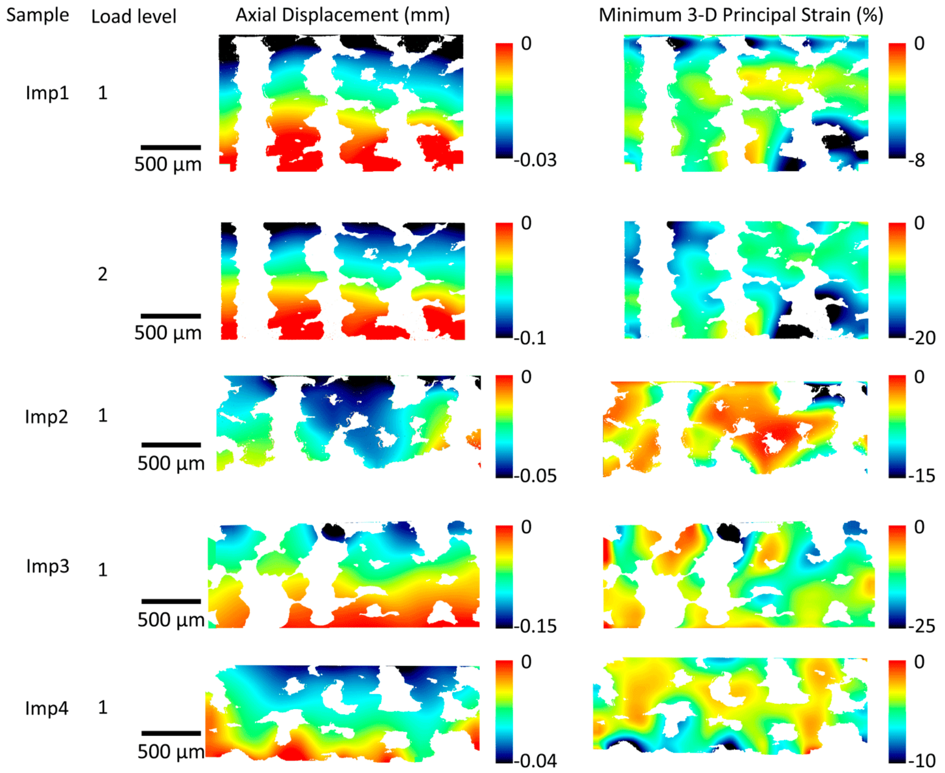

Figure S2. Displacement and strain calculated for thin hybrid implants using DVC. Shown here is one vertical slice through the centre of each 3D sample volume.

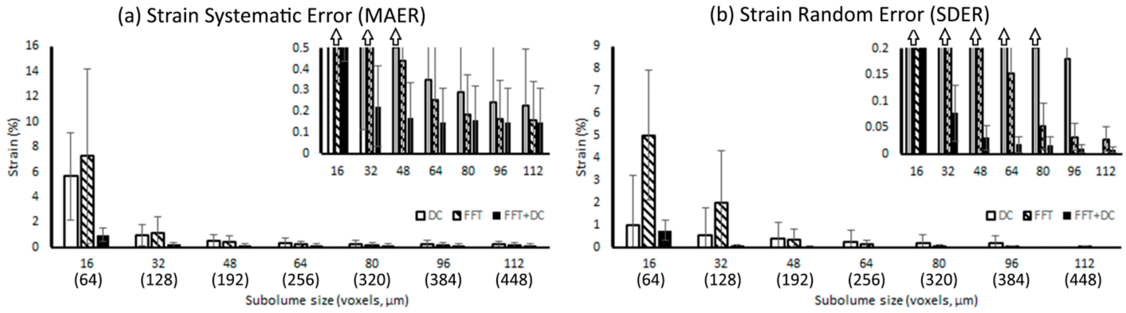

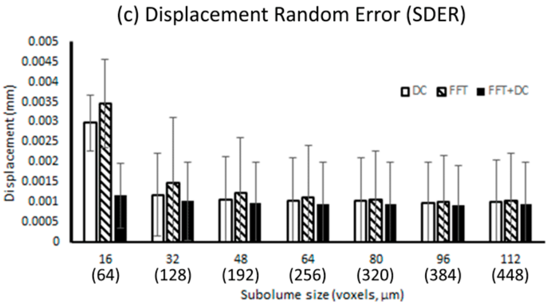

(d) DVC Error Summary

Metric	DVC Approach	Equation	R^2 Value	Value at chosen subvolume size (48 voxels)
Strain MAER	DC	$y = 1E+2x^{-2.015}$	0.9629	0.50%
	FFT	$y = 7.4075x^{-0.89}$	0.7767	0.44%
	FFT+DC	$y = 4E+1x^{-1.622}$	0.9461	0.17%
Strain SDER	DC	$y = 4E+1x^{-1.693}$	0.981	0.55%
	FFT	$y = 3E+2x^{-2.926}$	0.9675	0.33%
	FFT+DC	$y = 3E+1x^{-2.276}$	0.9669	0.03%
Displacement SDER	DC	$y = 0.0092x^{-0.506}$	0.7522	1.1 µm
	FFT	$y = 0.0141x^{-0.59}$	0.8582	1.2 µm
	FFT+DC	$y = 0.0015x^{-0.108}$	0.8896	0.97 µm

Figure S3. Quantification of error under constant-strain conditions using DVC. Strain systematic error (**a**) and random error (**b**) and displacement random error (**c**) for the hybrid scaffold samples (n = 6) for the three DVC approaches. DC = Direct Correlation, FFT = Fast Fourier transform, FFT+DC = combined approach of FFT and DC. Inset is the same data with the y-axis scaled to better show values for the larger subvolume sizes. The x axis is labelled in voxels and in brackets is the equivalent approximate micron distance valid for all scaffolds except Mech1 which utilised a larger voxel size. Error bars show the S.D. between all samples. Table (**d**) shows the power law equations and R2 values for each metric and DVC method.

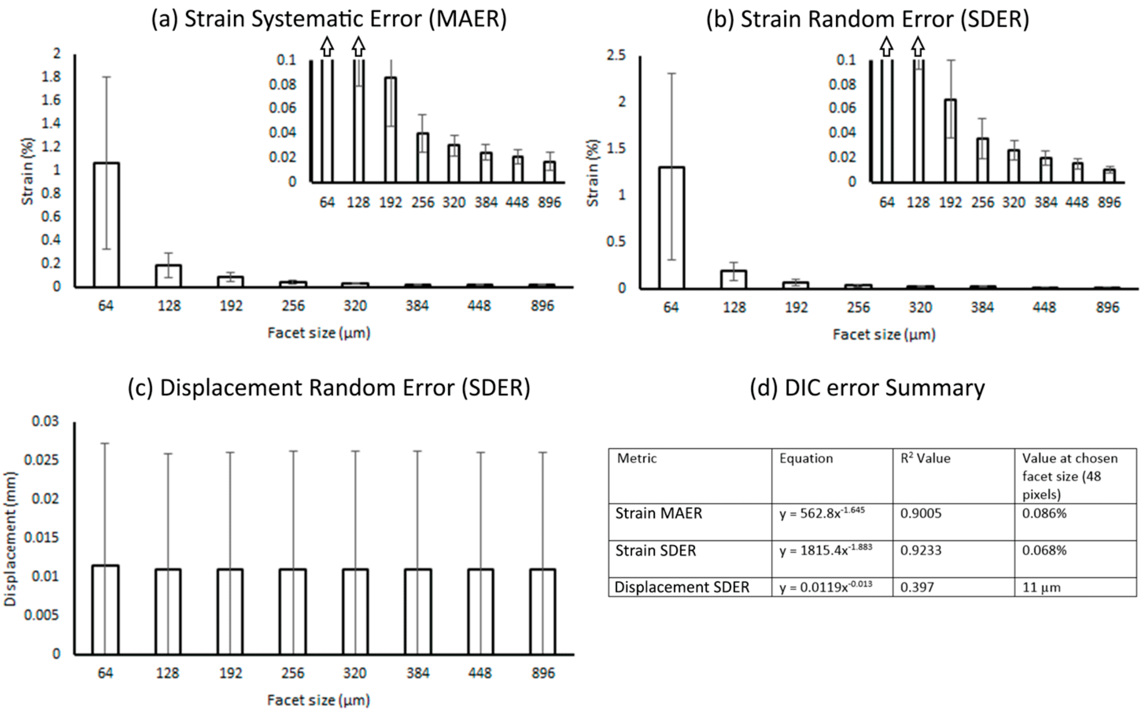

Metric	Equation	R^2 Value	Value at chosen facet size (48 pixels)
Strain MAER	$y = 562.8x^{-1.645}$	0.9005	0.086%
Strain SDER	$y = 1815.4x^{-1.883}$	0.9233	0.068%
Displacement SDER	$y = 0.0119x^{-0.013}$	0.397	11 µm

Figure S4. Quantification of error under constant-strain conditions using DIC. DIC analysis carried out on static photos of samples as a comparison to those collected during DVC and micro-CT constant strain error analysis for the hybrid scaffold samples (n = 6). (**a**) Strain systematic error (MAER), (**b**) Strain random error (SDER), (**c**) Displacement random error (SDER) and (**d**) Power law equations and R2 values for each metric. Error bars show the S.D. between all samples.

© 2020 by the authors. Submitted for possible open access publication under the terms and conditions of the Creative Commons Attribution (CC BY) license (http://creativecommons.org/licenses/by/4.0/).
